# Essential childbirth and postnatal interventions for improved maternal and neonatal health

**DOI:** 10.1186/1742-4755-11-S1-S3

**Published:** 2014-08-21

**Authors:** Rehana A Salam, Tarab Mansoor, Dania Mallick, Zohra S Lassi, Jai K Das, Zulfiqar A Bhutta

**Affiliations:** 1 Division of Women and Child Health, Aga Khan University, Karachi, Pakistan

**Keywords:** essential interventions, childbirth, immediate newborn care, infections, small and sick babies

## Abstract

Childbirth and the postnatal period, spanning from right after birth to the following several weeks, presents a time in which the number of deaths reported still remain alarmingly high. Worldwide, about 800 women die from pregnancy- or childbirth-related complications daily while almost 75% of neonatal deaths occur within the first seven days of delivery and a vast majority of these occur in the first 24 hours. Unfortunately, this alarming trend of mortality persists, as287,000 women lost their lives to pregnancy and childbirth related causes in 2010. Almost all of these deaths were preventable and occurred in low-resource settings, pointing towards dearth of adequate facilities in these parts of the world. The main objective of this paper is to review the evidence based childbirth and post natal interventions which have a beneficial impact on maternal and newborn outcomes. It is a compilation of existing, new and updated interventions designed to help physicians and policy makers and enable them to reduce the burden of maternal and neonatal morbidities and mortalities. Interventions during the post natal period that were found to be associated with a decrease in maternal and neonatal morbidity and mortality included: advice and support of family planning, support and promotion of early initiation and continued breastfeeding; thermal care or kangaroo mother care for preterm and/or low birth weight babies; hygienic care of umbilical cord and skin following delivery, training health personnel in basic neonatal resuscitation; and postnatal visits. Adequate delivery of these interventions is likely to bring an unprecedented decrease in the number of deaths reported during childbirth.

## Introduction

The number of deaths reported during childbirth and postnatal period still remain alarmingly high. Worldwide, about 800 women die from pregnancy- or childbirth-related complications daily and approximately 287,000 women lost their lives to pregnancy and childbirth related causes in 2010 [1]. Every year an estimated 2.9 million babies die in the first 4 weeks of life [2,3]. Almost all (99%) neonatal deaths occurs in low- and middle-income countries (LMICs), yet most epidemiological and other research focuses on mere 1% of deaths occurring in high income countries (HICs) [4]. The fact that almost all of these deaths were preventable and occurred in low-resource settings, points to a dearth of adequate facilities in these parts of the world. Preterm birth, birth asphyxia (lack of breathing at birth), and infections cause most neonatal deaths.The critical postpartum period starts from about an hour after the delivery of placenta and extends over the following six weeks. Postpartum care should respond to the special needs of the mother and baby during this critical period and should include prevention and early detection; treatment of complications and disease; attention to hygienic care; advice and support of exclusive breastfeeding; birth spacing; immunization; and maternal nutrition [5].

Since the postnatal period is a critical time to deliver interventions, failure to do so leads to detrimental effects on the survival and future health of both mother and neonate. Many women die as a result of complications during and following childbirth. The major complications that account for 80% of all maternal deaths are severe bleeding (mostly bleeding after childbirth), infections (usually after childbirth), high blood pressure during pregnancy (pre-eclampsia and eclampsia), and unsafe abortion. The remainder are caused by or associated with infectious diseases during pregnancy such as malaria and AIDS. A practical and viable strategy for reducing maternal and neonatal morbidity and mortality rates from preventable causes and meeting maternal health related Millennium Development Goal (MDG) targets, is by integrating improved childbirth facilities and postnatal care for newborns and mothers [6]. The 4^th^ MDG focuses on reducing child mortality and is closely associated with maternal health MDG. In developing countries alone, if mothers start practicing early initiation of breastfeeding, it is estimated that it can save as many as 1.45 million lives annually by reducing deaths mainly due to lower respiratory tract infections and diarrhoeal diseases [7]. To reduce the high burden of neonatal mortality and morbidity, postnatal care should be integrated into existing health programs. Community based education and health promotive workshops on exclusive breastfeeding and preventing vertical transmission of HIV will help increase the coverage of the postnatal interventions and improve maternal and newborn health.

This paper reviews and highlights the effectiveness of essential childbirth and immediate postnatal interventions for mothers and newborns during the parturition and postnatal period. This paper will help decision makers to deploy necessary interventions for improved maternal and newborn outcomes.

## Methodology

The methodology has been described in detail elsewhere [8]. In short, the review included all childbirth and postnatal interventions based on current World Health Organization (WHO) guidelines and recent Lancet series which have an alleged impact on reducing maternal, neonatal and child mortality; suitable for delivery in LMICs; and those that can be delivered through the health sector (community level up to the referral level of health care) (Table 1). All relevant childbirth and postnatal intervention reviews were identified from the electronic databases such as the Cochrane database of systematic reviews, the Cochrane database of abstract reviews of effectiveness (DARE), the Cochrane database of systematic reviews of randomized control trials (RCT’s), and PubMed. The reference lists of the reviews and recommendations from experts in the field were also used as sources to obtain additional publications. The principal focus was on the existing systematic reviews and meta-analysis. Based on the efficiency of the interventions, these were then classified in categories from A to E (where A signified strongly beneficial effect while E indicated harmful effect) on different levels of health sector (community/outreach/referral).

**Table 1:** Characteristics of the included reviews on childbirth and postnatal interventions

Reviews	Objective	Type of Studies included (number)	Cochrane/non-Cochrane	Pooled Data (Y/N)	Outcomes reported
**Hotnett 2013 **[10]	To assess the effects of continuous, one-to-one intrapartum support compared with usual care.	RCTs: 21	Cochrane	Yes	spontaneous vaginal birth, intrapartum analgesia, dissatisfaction, caesarean, instrumental vaginal birth, regional analgesia

**Smail 2010 **[16]	To assess the effects of prophylactic antibiotics compared with no prophylactic antibiotics on infectious complications in women undergoing cesarean section.	RCTs and qRCTs: 86	Cochrane	Yes	febrile morbidity, wound infection, endometritis and serious maternal infectious complications

**Cotter 2001 **[21]	To examine the effect of oxytocin given prophylactically in the third stage of labour on maternal and neonatal outcomes.	RCTs: 14	Cochrane	Yes	Blood loss, removal of placenta, blood pressure

**Soltani 2010 **[22]	To assess the effect of the timing of administration of prophylactic uterotonics (before compared to after placental delivery) on the outcomes related to the third stage of labour.	RCTs: 3	Cochrane	Yes	postpartum haemorrhage, retained placenta, length of third stage of labour, postpartum blood loss, changes in haemoglobin, blood transfusion; the use of additional uterotonics the incidence of maternal hypotension and the incidence of severe postpartum haemorrhage

**McDonald 2004 **[25]	To compare the effects of ergometrine-oxytocin with oxytocin in reducing the risk of PPH (blood loss of at least 500 ml) and other maternal and neonatal outcomes.	RCTs: 6	Cochrane	Yes	blood loss of at least 500 m

**Begley 2011 **[24]	To compare the effectiveness of active versus expectant management of the third stage of labour.	RCTs and qRCTs: 5	Cochrane	Yes	maternal primary haemorrhage, maternal haemoglobin

**McDonald 2013 **[26]	To determine the effects of early cord clamping compared with late cord clamping after birth on maternal and neonatal outcomes	RCTs:15	Cochrane	Yes	postpartum haemorrhage

**Pena-Marti 2007 **[27]	To determine the efficacy of fundal pressure versus controlled cord traction as part of the active management of the third stage of labour.	RCTs: 0	Cochrane	No	None

**Gulmezoglu 2012 **[30]	To evaluate the benefits and harms of a policy of labour induction at term or post-term compared to awaiting spontaneous labour or later induction of labour.	RCTs: 19	Cochrane	Yes	perinatal deaths, cesarean sections

**Hussain 2011 **[31]	The purpose of this review was to study the possible impact of induction of labour (IOL) for post-term pregnancies compared to expectant management on stillbirths.	Studies: 25 RCTs: 14	Non-Cochrane	Yes	Stillbirths

**Hofmeyr 2013 **[33]	To determine the effectiveness of uterine massage after birth and before or after delivery of the placenta, or both, to reduce postpartum blood loss and associated morbidity and mortality.	RCTs: 2	Cochrane	No	Blood loss

**Tuncalp 2012 **[34]	To assess the effects of prophylactic prostaglandin use in the third stage of labour.	RCTs: 72	Cochrane	Yes	severe PPH, blood transfusion

**Mousa 2007 **[35]	To assess the effectiveness and safety of pharmacological, surgical and radiological interventions used for the treatment of primary PPH	RCTs: 3	Cochrane	Yes	maternal mortality, hysterectomy, use of uterotonics, blood transfusion, or evacuation of retained products, maternal pyrexia

**Lopez 2010 **[39]	Assess the effects of educational interventions for postpartum mothers about contraceptive use	RCTs: 8	Cochrane	Yes	effect on contraceptive use

**Dodd 2004 **[45]	To assess the clinical effects of treatments for postpartum anaemia, including oral, intravenous or subcutaneous iron/folate supplementation and erythropoietin administration, and blood transfusion.	RCTs: 6	Cochrane	Yes	lactation at discharge from hospital

**French 2004 **[46]	The effect of different antibiotic regimens for the treatment of postpartum endometritis on failure of therapy and complications was systematically reviewed.	RCTs: 47	Cochrane	Yes	treatment failures

**Kesho Bora 2009 **[47]	Triple-antiretroviral (ARV) prophylaxis during pregnancy and breastfeeding compared to short-ARV prophylaxis to prevent mother-to-child transmission of HIV-1 (PMTCT): the Kesho Bora randomized controlled clinical trial in five sites in Burkina Faso, Kenya	1 study in five different location	Non Cochrane	No	Extended triple ARV regimen consisting of the anti-HIV drugs zidovudine, lamivudine andlopinavir/ritonavir, from the last trimester of pregnancy and continued during breastfeeding up to the age of six months.

**McCall 2010 **[49]	To assess efficacy and safety of interventions designed for prevention of hypothermia in preterm and/or low birthweight infants applied within ten minutes after birth in the delivery suite compared with routine thermal care.	RCTs: 6	Cochrane	Yes	heat losses in infants < 28 weeks' gestation, risk of death within hospital stay

**Dyson 2005 **[52]	To evaluate the effectiveness of interventions which aim to encourage women to breastfeed in terms of changes in the number of women who start to breastfeed.	RCTs: 7	Cochrane	Yes	increasing breastfeeding initiation rates

**Lewin 2010 **[53]	To assess the effects of LHW interventions in primary and community health care on maternal and child health and the management of infectious diseases.	RCTs: 82	Cochrane	Yes	increasing breastfeeding initiation rates

**Lassi 2010 **[54]	To assess the effectiveness of community-based intervention packages in reducing maternal and neonatal morbidity and mortality; and improving neonatal outcomes.	RCTs and qRCTs: 18	Cochrane	Yes	Maternal mortality, neonatal mortality, perinatal morality, stillbirths, newborn care practices

**Imdad 2011 **[55]	To assess the effectiveness of breastfeeding promotion interventions on breastfeeding rates in early infancy.	RCTs and qRCTs: 53	Non-Cochrane	Yes	EBF at 4-6 weeks postpartum

**Debes 2013 **[56]	To review the evidence for early breastfeeding initiation practices and to estimate the associationbetween timing and neonatal outcomes.	prospective studies,includingRCTs, and cohort studies = 18	Non-Cochrane	Yes	All-cause neonatal mortality, infection-related neonatal mortality

**Lumbiganon 2011 **[57]	To evaluate the effectiveness of antenatal BF education for increasing BF initiation and duration.	RCTs: 17	Cochrane	No	BF educational interventions were not significantly better than a single intervention

**Imdad 2013 **[62]	To evaluate the effects of application of chlorhexidineto the umbilical cord to children born in low income countries on cord infection (omphalitis) and neonatal mortality.	3 RCTs	Non-Cochrane	Yes	All cause neonatal mortality, omphalitis

**Zupan 2004 **[60]	To assess the effects of topical cord care in preventing cord infection, illness and death.	RCTs and qRCTs: 21	Cochrane	Yes	colonization with antibiotics

**Ziino 2002 **[67]	To determine if the administration of epinephrine to apparently stillborn and extremely bradycardic newborns reduces mortality and morbidity	RCTs: 0	Cochrane	No	-

**Lee 2011 **[68]	To estimate the mortality effect of immediate newborn assessment and stimulation, and basic resuscitation on neonatal deaths due to term intrapartum-related events or preterm birth, for facility and home births.	RTs: 2 qRCT: 2 Observational studies: 20	Non-Cochrane	Yes	preterm birth

**Ungerer 2004 **[69]	To assess the effect of prophylactic versus selective antibiotic treatment for asymptomatic term neonates born to mothers with risk factors for neonatal infection.	RCTs: 2	Cochrane	No	-

**Mtitimila 2004 **[72]	To compare effectiveness and adverse effects of antibiotic regimens for treatment of presumed early neonatal sepsis.	RCTs: 2	Cochrane	Yes	Mortality, treatment failure or bacteriological resistance.

**Gordon 2005 **[73]	To compare the effectiveness and adverse effects of different antibiotic regimens for treatment of suspected late onset sepsis in newborn infants.	RCTs:13	Cochrane	No	Mortality, treatment failure

**Sazawal 2003 **[74]	This meta-analysis provides estimates of mortality impact of the case-management approach proposed by WHO.	RCTs: 7	Non-Cochrane	Yes	All-cause mortality, pneumonia specific mortality

**Zaidi 2011 **[75]	We conducted systematic searches of multiple databases to identify relevant studies with mortality data.	RCTs: 7	Non-Cochrane	Yes	All-cause mortality, pneumonia specific mortality

**Bhutta 2009 **[76]	We reviewed available evidence for community-based antibiotic management strategies for serious neonatal infections.	RCTs:9	Non-Cochrane	Yes	All-cause mortality, pneumonia specific mortality

**Lawn 2010 **[79]	to review the evidence, and estimate the effect of KMC on neonatal mortality due to complications of preterm birth.	RCTs:9 Observational studies: 5	Non-Cochrane	Yes	neonatal mortality

**Conde-Agudelo 2011 **[80]	To determine whether there is evidence to support the use of KMC in LBW infants as an alternative to conventional care after the initial period of stabilization with conventional care.	RCTs:3	Cochrane	Yes	nosocomial infection, severe illness, lower respiratory tract, not exclusively breastfeeding at discharge, and maternal dissatisfaction

**Edmond 2006 **[84]	This review summarizes the evidence on feeding LBW infants and serves as the basis for the development of guidelines on feeding LBW infants in developing countries.	Systematic reviews, RCTs, observational studies and descriptive studies	Non-Cochrane	No	What to feed and optimal duration of breastfeeding

**Soll 2009 **[86]	To determine the effect of multiple doses of exogenous surfactant compared to single doses of exogenous surfactant on mortality and complications of prematurity in premature infants at risk for or having respiratory distress syndrome.	RCTs:3	Cochrane	Yes	pneumothorax and risk of mortality

**Soll 1998 **[92]	o assess the effect of intratracheal administration of synthetic surfactant in premature newborns with established respiratory distress syndrome (RDS).	RCTs:6	Cochrane	Yes	Pneumothorax, pulmonary interstitial emphysema, patent ductusarteriosus, risk of intraventricular hemorrhage, risk of bronchopulmonary dysplasia, risk of neonatal mortality

**Soll 2012 **[93]	To compare the effects of early vs. delayed selective surfactant therapy for newborns intubated for respiratory distress within the first two hours of life. Planned subgroup analyses include separate comparisons for studies utilizing natural surfactant extract and synthetic surfactant.	RCTs:4	Cochrane	Yes	pneumothorax and pulmonary interstitial emphysema

**Greenough 2008 **[98]	To compare the efficacy of: (i) synchronized mechanical ventilation, delivered as high frequency positive pressure ventilation (HFPPV) or patient triggered ventilation - assist control ventilation (ACV) or synchronous intermittent mandatory ventilation (SIMV)) with conventional ventilation (CMV) (ii) different types of triggered ventilation (ACV, SIMV, pressure regulated volume control ventilation (PRVCV) and SIMV plus pressure support (PS)	RCTs:14	Cochrane	Yes	Air leak, duration of ventilation, duration of weaning

**Lemyre 2002 **[99]	In preterm infants with recurrent apnea, does treatment with NIPPV lead to a greater reduction in apnea and need for intubation and mechanical ventilation, as compared with treatment with NCPAP? Does NIPPV increase the incidence of gastrointestinal complications, i.e. gastric distension leading to cessation of feeds, or perforation?	RCTs:2	Cochrane	Yes	rates of apnea

**Ho 2002 **[100]	In spontaneously breathing preterm infants with RDS, to determine if continuous distending pressure (CDP) reduces the need for IPPV and associated morbidity without adverse effects	RCTs:6	Cochrane	No	blood used for exchange transfusion

**Thayyil 2006 **[102]	To compare the effectiveness of single volume exchange transfusion (SVET) with that of double volume exchange transfusion (DVET) in producing survival without disability and reducing bilirubin levels in newborn infants with severe jaundice.	RCTs:1	Cochrane	Yes	blood used for exchange transfusion

**Mills 2001 **[101]	To evaluate the efficacy of fibreoptic phototherapy.	RCTs:31	Cochrane	Yes	serum bilirubin

### Childbirth interventions

#### Social support during childbirth

Historically and cross-culturally, women have been attended and supported by other women during childbirth. However, since the middle of the 20th century, in many countries as the majority of women gave birth in hospital rather than at home, continuous support during labour has become an exception rather than the routine. Concerns about dehumanization of women’s birth experiences (in high-, middle-, and low income countries) have led to calls for a return to continuous, one-to-one support by women for women during labour [9]. Common elements of this care include emotional support (continuous presence, reassurance and praise), information about labour progress and advice regarding coping techniques, comfort measures (such as comforting touch, massage, warm baths/showers, promoting adequate fluid intake and output) and advocacy (helping the woman articulate her wishes to others).

A systematic review by Hodnett et al. reported that women allocated to continuous support were more likely to have a spontaneous vaginal birth (risk ratio (RR) 1.08, 95% confidence interval (CI) 1.04, 1.12) and less likely to have intrapartum analgesia (RR 0.90, 95% CI 0.84, 0.97) or report dissatisfaction (RR 0.69, 95% CI 0.59, 0.79). In addition their labour were shorter (mean difference (MD) -0.58 hours, 95% CI -0.86, -0.30), they were less likely to have a caesarean (RR 0.79, 95% CI 0.67, 0.92) or instrumental vaginal birth (RR 0.90, 95% CI 0.84, 0.96), regional analgesia (RR 0.93, 95% CI 0.88, 0.99), or a baby with a low 5-minute Apgar score (RR 0.70, 95% CI 0.50, 0.96) [10]. Continuous social support has shown significant clinical benefits for women and infants. All women should receive continuous social support throughout labour and childbirth.

#### Prophylactic antibiotic for caesarean sections

The single most important risk factor for post-partum maternal infection is caesarean delivery [11]. Women undergoing caesarean section have a 5 to 20-fold greater risk for infection compared with a vaginal delivery. Infectious complications that occur after caesarean delivery are an important and substantial cause of maternal morbidity and are associated with a significant increase in hospital stay [12]. Infectious complications following caesarean delivery include fever, wound infection, endometritis, bacteraemia, other serious infection (including pelvic abscess, septic shock, necrotizing fasciitis and septic pelvic vein thrombophlebitis) and urinary tract infection [11,13,14]. Fever can occur after any operative procedure and a low grade fever following a caesarean delivery may not necessarily be a marker of infection [15].

Prophylactic antibiotics in women undergoing caesarean section substantially reduce the incidence of febrile morbidity (RR 0.45; 95% CI 0.39, 0.51), wound infection (RR 0.39; 95% CI 0.32, 0.48), endometritis (RR 0.38; 95% CI 0.34, 0.42) and serious maternal infectious complications (RR 0.31; 95% CI 0.19, 0.48) [16]. Based on these findings, we would recommend prophylactic antibiotics for women undergoing elective or non-elective caesarean sections however further studies are needed to explore potential adverse effects.

#### Prevention of postpartum haemorrhage

Excessive bleeding or postpartum haemorrhage (PPH) during or after childbirth is a life-threatening complication and a major contributor to maternal mortality worldwide [17]. Primary PPH is defined as heavy bleeding directly following childbirth or within 24 hours of thereafter [18]. One of the methods by which PPH is prevented is by active management of third stage of labour [19], while others include measures like uterine massage. Active management of third stage of labour (AMTSL) has three components which include the use of uterotonic agents, early cord clamping and controlled cord contraction. In 2012, the results of a large WHO-directed, multi-centre clinical trial [20] showed that administration of the uterotonic was the most important AMTSL component. This trial also demonstrated that the addition of Controlled Cord Traction had minimum effect on the reducing haemorrhage. Carbetocin (oxytocin) is a long acting synthetic analogue of oxytocin with pharmacological, clinical and agonist properties similar to oxytocin. It acts by combining to oxytocin receptors present in the uterus and causes increased tone and rhythmic contractions of the uterus and also increases the frequency of existing contractions.

i) Oxytocin for PPH

Prophylactic oxytocin showed benefits in reducing blood loss >500ml (RR 0.50; 95% CI 0.43, 0.59) and need for therapeutic oxytocics (RR 0.50; 95% CI 0.39, 0.64) compared to no uterotonic [21]. While another review showed no influence of oxytocin before and after the expulsion of placenta on incidence of PPH (blood loss >500ml) (RR 0.81; 95% CI 0.62, 1.04); retained placenta (RR 1.54, 95% CI 0.76, 3.11); length of third stage of labour (minutes) (Mean difference (MD) -0.30, 95% CI -0.95, 0.36); postpartum blood loss (ml) (MD 22.32, 95% CI -58.21, 102.86); changes in haemoglobin (g/dL) (MD 0.06, 95% CI -0.60, 0.72); blood transfusion (RR 0.79, 95% CI 0.23, 2.73); use of additional uterotonic (RR 1.10, 95% CI 0.80, 1.52); incidence of maternal hypotension (RR 2.48, 95% CI 0.23, 26.70) and incidence of severe PPH (blood loss ≥1000 ml) (RR 0.98, 95% CI 0.48, 1.98) [22].

i) Active management of third stage of labour

In this section we included reviews that compared AMTSL with expectant management (when the placenta is allowed to deliver spontaneously or aided by gravity and nipple stimulation). According to the latest guideline of WHO, AMTSL as a prophylactic intervention is composed of three steps: 1) administration of a uterotonic, preferably oxytocin, immediately after birth of the baby; 2) controlled cord traction (CCT) to deliver the placenta; and 3) massage of the uterine fundus after the placenta is delivered. In 2012, the results of a large WHO-directed, multi-centre clinical trial [20] showed that the most important AMTSL component was the administration of the uterotonic. In view of the data from this trial and the existing evidence concerning the role of routine uterine massage in the prevention of PPH, the WHO issued new recommendations which state that although administration of a uterotonic remains central to the implementation of AMTSL, the performance of CCT and immediate fundal massage are optional components [23].

Active management of third stage of labor showed reduction in the risk of haemorrhage at time of birth (>1000ml) (RR 0.34, 95% CI 0.14, 0.87) and of maternal haemoglobin (Hb) <9g/dL following birth (RR 0.50, 95% CI 0.30, 0.83) [24]. Compared with oxytocin, ergometrine-oxytocin was associated with a small reduction in the risk of PPH (OR 0.82, 95% CI 0.71, 0.95) [25]. On the other hand, no significant differences were found between early versus late cord clamping on severe PPH (RR 1.04, 95% CI 0.65, 1.65) or for PPH (>500ml) (RR 1.17 95% CI 0.94, 1.44) [26]. Another review by Pena-Marti et al. did not find studies that compared fundal pressure versus controlled cord traction as part of AMSTL [27]. These findings suggest active management is superior to expectant management in terms of reduction in the risk of maternal haemorrhage at time of birth. However, statistically significant results in the improved outcomes of blood loss and PPH could not be found when active management was employed. On the other hand, prophylactic oxytocin showed benefits in reducing blood loss >500ml and need for therapeutic oxytocics.

#### Induction of prolonged pregnancy

Five to ten percent of pregnancies continue beyond 294 days (42 completed weeks) and are described as being ‘post-term’ or ‘postdate’ [28]. Both the mother and the infant are at increased risk of adverse events when the pregnancy continues beyond term [29]. Hilder et al. 1998 reported that after 41weeks, neonatal and post neonatal death risk increased significantly. The obstetric problems associated with post-term pregnancy include induction of labour with an unfavourable cervix, caesarean section, prolonged labour, PPH and traumatic birth. It is likely that some of these unwanted outcomes result from intervening when the uterus and cervix are not ready for labour [29].

Compared with a policy of expectant management, a policy of labour induction was associated with fewer (all-cause) perinatal deaths (RR 0.31, 95% CI 0.12, 0.88). The number needed to treat to benefit (NNTB) with induction of labour in order to prevent one perinatal death was 410 (95% CI 322, 1492) [30]. Another meta-analysis of trials also suggested that a policy of elective induction of labour for pregnancies at or beyond 41 weeks is associated with significantly fewer perinatal deaths (RR 0.31; 95% CI: 0.11, 0.88) compared to expectant management, but no significant difference in the incidence of stillbirth (RR 0.29; 95% CI: 0.06, 1.38) was noted. There was significant decrease in incidence of neonatal morbidity from meconium aspiration (RR 0.43, 95% CI 0.23, 0.79) and macrosomia (RR 0.72; 95% CI: 0.54, 0.98) [31]. In case of absence of a specific disorder, induction of labour can be proposed in patients between 41(+0) and 42(+6) weeks (grade B). It is important to convey to the decision makers and patients that the choice of prolonging beyond 42(+0) weeks appears to involve an increase risk and decision should be weighed and balanced against the potential disadvantages of induction (Professional consensus). Stripping the membranes can reduce the duration of pregnancy by increasing the number of patients going into labour spontaneously during the week afterward (grade B) [32]. This policy is associated with fewer deaths. There does not seem to be any increased risk of assisted vaginal or abdominal delivery. If the woman chooses to wait for spontaneous labour onset it would be prudent to have regular fetal monitoring as longitudinal epidemiological studies suggest increased risk of perinatal death by increasing gestational age.

#### Management of postpartum haemorrhage

Primary PPH requires a multidisciplinary approach. First line treatment is with uterotonic (such as ergometrine, oxytocin, prostaglandins) accompanied with bimanual compression of the uterus. The second line therapy is surgical, usually hysterectomy to prevent death from uterine haemorrhage.

i) Uterine massage

The review by Hofmeyr et al. included two randomised controlled trials [33]. The first trial randomised women to receive uterine massage or no massage following delivery of the placenta, after active management of the third stage of labour including use of oxytocin. The numbers of women with blood loss >500ml was small, with no difference (RR 0.52, 95% CI 0.16, 1.67) while the mean blood loss was significantly less in the uterine massage group at 30 minutes (MD -41.60ml, 95% CI -75.16, -8.04) and 60 minutes (MD -77.40ml, 95% CI -118.71, -36.09). There were no cases of retained placenta in either group.. The need for additional uterotonic was also significantly reduced in the uterine massage group (RR 0.20, 95% CI 0.08, 0.50). For use of uterine massage before and after delivery of the placenta, another trial assigned women to receive oxytocin, uterine massage or both after delivery of the baby but before delivery of the placenta. There was no added benefit for uterine massage plus oxytocin over oxytocin alone with respect to blood loss ≥500ml (RR 1.56, 95% CI 0.44, 5.49) or need for additional use of uterotonic (RR 1.02, 95% CI 0.56, 1.85) [33].

ii) Use of uterotonic

Oral or sublingual misoprostol when compared with placebo was found to be effective in reducing severe PPH (sublingual: RR 0.66; 95% CI 0.45, 0.98) and blood transfusion (oral: RR 0.31; 95% CI 0.10, 0.94). Compared with conventional injectable uterotonic, oral misoprostol was associated with higher risk of severe PPH (RR 1.33; 95% CI 1.16, 1.52) and use of additional uterotonic, but with a trend towards fewer blood transfusions (RR 0.84; 95% CI 0.66, 1.06) [34]. The comparison of misoprostol (dose 600-1000 mcg) with placebo did not show any impact on reducing maternal mortality (RR 7.24, 95% CI 0.38, 138.6), hysterectomy (RR 1.24, 95% CI 0.04, 40.78), additional use of uterotonic (RR 0.98, 95% CI 0.78, 1.24), blood transfusion (RR 1.33, 95% CI 0.81, 2.18), or evacuation of retained products (RR 5.17, 95% CI 0.25, 107). Misoprostol use was associated with a significant increase in maternal pyrexia (RR 6.40, 95% CI 1.71, 23.96) and shivering (RR 2.31, 95% CI 1.68, 3.18) [35]. These findings suggest that misoprostol is effective in reducing severe PPH and blood transfusion. In comparison with conventional injectable uterotonic, oral misoprostol was associated with higher risk of severe PPH and use of additional uterotonic, but with a trend to fewer blood transfusions.

### Postnatal (mother)

#### Advice and provision of family planning

The provision of contraceptive education is now considered a standard component of postpartum care. Family planning advice and education about contraception are commonly provided to women who have just given birth and is frequently provided as part of discharge planning [36]. Decisions about contraception made right after counseling may differ considerably from contraceptive use postpartum [37]. As common as postpartum contraceptive education has become, research to evaluate such interventions is still sparse [38].

Oral contraceptive education program (one time) versus routine counselling led to a decrease in the incidence of known pregnancy by one year (odds ratio (OR) 0.81; 95%CI: 0.11, 6.04), increase in the rate of continuation of oral contraceptives at one year (OR 0.67; 95% CI: 0.11, 3.99) and a greater chance of switched contraceptives by one year(OR 2.0; 95% CI: 0.37, 10.92) [39]. Contraceptive counseling (one time) versus no counseling led to an increase in the use of any contraceptive at 8 to12 weeks postpartum (OR 19.56; 95% CI: 11.65, 32.83) and an increase in the choice of modern contraceptive (using or plan to use) at 8 to 12 weeks postpartum (OR 1038.09; 95% CI: 64.15,16799.73) [39]. Health education including contraception with an immediate session versus no immediate session increased the use of contraception at 3 months (OR 1.50; 95% CI: 0.88, 2.54) and at 6 months (OR 1.62; 95% CI: 1.06, 2.50) [39]. After a later session versus no session, use of contraception at 6 months did not increase significantly (OR 0.95; 95% CI: 0.50, 1.80). Special postpartum care (including contraception) versus routine services (multiple well-baby contacts) led to decreased incidence of a repeat pregnancy (self-report) by 18 months (OR 0.35; 95% CI: 0.17, 0.70) [39]. These findings suggest that postpartum education about contraception leads to more contraception use and fewer unplanned pregnancies. Such education may be effective in increasing the short-term use of contraception. However, there are only limited data examining a more important longer-term effect on the prevention of unplanned pregnancies.

#### Detection and treatment of maternal anaemia

Anaemia after the birth of a baby (postpartum anaemia) is a common problem throughout the world and for most women is self-limiting, resolving within a week [40]. For some women however, particularly in resource-poor countries, it is a major cause of maternal morbidity (poor health) and mortality [41-43]. In this setting, anaemia may result from inadequate dietary intake, parasitic infection or malaria, and may be exacerbated by the physiological effects of pregnancy and blood loss at the time of birth [44]. Traditional treatments include iron supplementation and blood transfusion for severe anaemia. A hormone, erythropoietin, may help improve iron levels in the blood and the woman’s ability to lactate.

When compared with iron therapy only, erythropoietin increases the likelihood of lactation at discharge (RR 1.90; 95% CI: 1.21, 2.98) and decrease the need for blood transfusion (RR 0.20; 95% CI: 0.01, 3.92) [45].However, the review did not identify any trial that assessed treatment with blood transfusion. Currently, limited evidence exists for the treatment of anaemia with erythropoiten and high quality trails are needed to assess the treatment of postpartum anaemia with iron supplementation and blood transfusion.

#### Detection and management of postpartum sepsis

Inflammation of the lining of the womb (postpartum endometritis), also known as puerperal fever, is caused by infection entering the womb (uterus) during childbirth. It occurs in about 1 to 3% of births, and is up to ten times more common after caesarean section. Prolonged rupture of membranes and multiple vaginal examinations also increase the risk. Endometritis causes fever, uterine tenderness and unpleasant-smelling lochia, and it can have serious complications such as abscess formation, sepsis and blood clots. It is also an important cause of maternal mortality worldwide, although this is very rare in HICs with the use of antibiotics.

Fifteen studies comparing clindamycin and aminoglycoside with another regimen found more treatment failures with the other regimen (RR 1.44; 95% CI 1.15, 1.80). Failures of those regimens with poor activity against penicillin resistant anaerobic bacteria were more likely (RR 1.94; 95% CI 1.38, 2.72) [46]. The combination of gentamicin and clindamycin appears suitable for the treatment of endometritis while regimens with activity against penicillin resistant anaerobic bacteria are better than those without them.

#### Screening and initiation or continuation of antiretroviral therapy for HIV

Antiretroviral (ARV) drugs reduce viral replication and can reduce mother-to-child transmission of HIV either by lowering plasma viral load in pregnant women or through post-exposure prophylaxis in their newborns. In HICs, highly active antiretroviral therapy (HAART) which usually comprises three drugs has reduced the mother-to-child transmission rates to around 1-2%. Recent evidence published in Lancet Infectious Diseases based on the Kesho Bora Studies shows that giving a combination of three ARV drugs to pregnant mothers with HIV infection from the last trimester, through delivery and six months of breastfeeding reduces the risk of transmitting HIV to the baby and improves survival. Infants of mothers whose virus is fully suppressed (undetectable) by triple-ARVs at the time of delivery have a very low risk of HIV infection (only 2.7% by the age of one year).It is therefore important to start ARVs early in pregnancy, ideally before pregnancy, for all women who require ART (CD4 count at or below 350 cells/mm3).

The Kesho Bora study shows that a significant reduction in infant infection can be achieved when pregnant women with a CD4 immune cell count of 200-500 cells/ mm3 are given a combination of three ARVs to prevent transmission. This treatment should start in their last trimester of pregnancy, continuing through birth and six months of breastfeeding. This was shown to reduce the risk of transmitting HIV to the baby and improved survival compared with babies of mothers with HIV who are given the current WHO-recommended short-course ARV regimen in late pregnancy and around the time of delivery [47]. These findings suggest that a regimen combining triple ARV is most effective for preventing transmission of HIV from mothers to babies. The risk of adverse events to both mother and baby appears low in the short-term but the optimal antiretroviral combination and the optimal time to initiate this to maximize prevention efficacy without compromising the health of either mother or baby remains unclear.

### Immediate essential newborn care

#### Thermal care for preterm babies and/or low birth weight infants

An optimal thermal environment is desirable for preterm infants. When an infant is challenged by cold, the baby attempts to conserve body heat by vasoconstriction and to maintain body temperature via thermogenesis by the metabolism of brown adipose tissue and an increase in oxygen consumption. The increase in energy expenditure may reduce weight gain [48]. A number of measures have been suggested to assist in the maintenance of body temperature for infants except the traditionally used incubators.

The intervention includes (1) barriers to heat loss applied to any part of the body of the preterm and/or low birth weight (LBW) infant within 10 minutes after birth in the delivery suite. These would consist of coverings such as transparent plastic wraps and bags made of low-density polyethylene (LDPE) or semi-permeable membranes such as opsite or Tegaderm and other additional swaddling materials or wraps (excluding delivery room blankets) such as the ’silver swaddler’; 2) External heat sources (non-routine) initiated within 10 minutes after birth in the delivery suite such as skin-to-skin care and heated/gel/chemical mattresses. Plastic wraps or bags were found to be effective in reducing heat losses in infants <28 weeks' gestation (Weighted Mean Difference (WMD) 0.68°C; 95% CI 0.45, 0.91), but not in infants between 28 to 31 week's gestation. Plastic caps were effective in reducing heat losses in infants <29 weeks' gestation (MD 0.80°C; 95% CI 0.41, 1.19). There was insufficient evidence to suggest that either plastic wraps or plastic caps reduce the risk of death within hospital stay. On the other hand, skin-to-skin care was shown to be effective in reducing the risk of hypothermia when compared to conventional incubator care for infants (RR 0.09; 95% CI 0.01, 0.64). The transwarmer mattress reduced the incidence of hypothermia on admission to NICU in very LBW infants (RR 0.30; 95% CI 0.11, 0.83) [49]. These findings suggest that any intervention other than primary care designed for prevention of hypothermia, and applied within 10 minutes after birth in the delivery suite, may be beneficial in practice including plastic wraps and bags, skin-to-skin contact, and transwarmer mattresses. These interventions keep infants warmer and decreased the incidence of hypothermia. To prevent the morbidity and mortality in preterm infants due to hypothermia, consideration should be given to using these interventions in the delivery suite.

#### Advise on support of early initiation of breastfeeding

The benefits of breastfeeding on mother and newborn have been well documented, and the WHO recommends that all mothers initiate breastfeeding within the first hour after giving birth [50,51]. Timing of start of breastfeeding is very important, with studies suggesting much greater benefits of early versus late feeding. Early initiation of breastfeeding is defined as feeding within the first day after delivery while late (delayed) initiation of breast feeding is when it starts after the first day of delivery.

A review of interventions to promote breastfeeding showed increased rates of initiation of breastfeeding (RR 1.53; 95% CI: 1.25, 1.87) [52]; another review also showed similar results (RR 1.45; 95% CI: 1.14, 1.84) [53]. Community-based intervention packages for reducing maternal and neonatal morbidity and mortality also showed an increase in rates of breastfeeding (RR 1.94; 95% CI: 1.56, 2.42) [54]. Early versus late initiation of breastfeeding was found to be associated with decreased neonatal mortality (RR 0.52; 95% CI: 0.27, 0.98) while the effect of breastfeeding when compared to no breastfeeding also decreased neonatal mortality (RR 0.30; 95% CI: 0.17, 0.53) [55]. Another review showed that early breastfeeding initiation was associated with lower risks of all-cause neonatal mortality among all live births (RR 0.56; 95% CI: 0.40, 0.79) and among LBW babies (RR 0.58; 95% CI: 0.43, 0.78), as well as infection-related neonatal mortality (RR 0.55; 95% CI: 0.36, 0.84) [56]. When evaluating antenatal breastfeeding education, a formal breastfeeding education workshop vs. routine care increased the initiation rate of breastfeeding (RR 1.19; 95% CI: 0.97, 1.45); peer counseling versus routine care showed higher increments in the initiation rates of breastfeeding (RR 1.82; 95% CI: 1.13, 2.93). Less significant increases were observed at 3 months and 6 months after the education workshop [57]. Another review by Imdad et al. 2011 compared breastfed versus non breastfed infants and showed a significant 70% reduction in the risk of neonatal mortality. It also showed that early initiation of breastfeeding versus late reduced neonatal mortality significantly by 48%.

#### Promotion and provision of hygienic cord and skin care

An umbilical cord infection may be clinically obvious while sometime tracking of bacteria along the umbilical vessels might not be apparent but can lead to septicemia, or other focal infections such as septic arthritis as a result of blood-borne spread [58,59]. In such cases, affected babies may also present with fever, lethargy or poor feeding. Soon after a normal delivery, the skin of the newborn baby including the umbilical stump is colonized mainly by nonpathogenic bacteria such as coagulase negative Staphylococci and Diphtheria bacilli. Pathogenic bacteria such as Coliforms and Streptococci may also be present on the skin and can track up the umbilical stump causing infection. It is therefore essential to keep the cord clean.

Antiseptic versus dry cord care/placebo decreased cord infection rates (RR 0.53; 95% CI: 0.25, 1.13); alcohol showed lower cord infection rates as well (RR 0.63; 95% CI: 0.19, 2.06]; triple dye also lowered infection (RR 0.68; 95% CI: 0.13, 3.49]; salicylic sugar powder showed significant reductions in infection rates (RR 0.21; 95% CI: 0.01, 4.38). Antiseptics that were aqueous based and alcohol based were effective for cord separation: (WMD -4.76; 95% CI: -5.34, -4.19) and (WMD -10.05; 95% CI: -10.72, -9.38) respectively, when compared with powder based antiseptics [60]. A single study [61] evaluating the effect of chlorhexidine cleansing of the newborn skin showed reduced rate of bacterial colonization by Staphylococcus aureus (RR 0.65; 95%CI: 0.55, 0.77), Streptococci (RR 0.53; 95%CI: 0.27, 1.04); and E. Coli infections [61]. Chlorhexidine versus no treatment in cleaning skin of LBW newborn decreased neonatal mortality (RR 0.72; 95%CI: 0.55, 0.95) [61]. Another review also indicated that the use of chlorhexidine led to a 23% reduction in all-cause neonatal mortality in the intervention group compared to control (RR 0.77; 95 % CI: 0.63, 0.94) [62]. A study conducted in Nepal found that there was a 28% reduction in mortality among LBW infants when chlorhexidine was used to clean the skin [63].

#### Neonatal resuscitation with bag and mask for babies who do not breathe at birth

At birth, the lungs of newborn babies are filled with fluid. This fluid must be cleared and replaced with air after birth. While most babies manage by themselves, one in every 20 to 30 newborns receives resuscitation at birth, mostly for absent or ineffective breathing. Effective ventilation is the key to successful neonatal resuscitation. Positive pressure ventilation is initiated with manual ventilation devices which may or not deliver positive end-expiratory pressure (PEEP).

A review by OO'Donnell et al. found insufficient evidence to determine the efficacy and safety of PEEP during positive pressure ventilation at neonatal resuscitation [64]. Ventilation is frequently initiated with a manual resuscitation bag and face-mask (BMV) followed by endotracheal intubation (ETT) if depression continues. These techniques may be difficult to perform successfully resulting in prolonged resuscitation or severe neonatal depression. The laryngeal mask airway (LMA) may achieve initial ventilation and successful resuscitation faster than a bag-mask device or endotracheal intubation. The review by Grein et al. found no difference between the LMA and ETT with the exception of a clinically insignificant difference in time to complete insertion of the device favoring the ETT [65]. Recent American Academy of Pediatrics guidelines reported that LMA that fit over the laryngeal inlet have been shown to be effective for ventilating newborns weighing more than 2000 g. A laryngeal mask should be considered during resuscitation if facemask ventilation is unsuccessful and tracheal intubation is unsuccessful or not feasible. While, endotracheal intubation may be indicated for initial endotracheal suctioning of non-vigorous meconium-stained newborns, if bag-mask ventilation is ineffective or prolonged, when chest compressions are performed or for special resuscitation circumstances, such as congenital diaphragmatic hernia or extremely low birth weight [66]. Despite formal guidelines for the use of epinephrine in neonatal resuscitation, the evidence for these recommendations has not yet been rigorously scrutinized. A review evaluating the effectiveness of epinephrine in the context of apparent stillbirth or extreme bradycardia found no studies meeting the criteria for inclusion [67]. A meta-analysis of three facility-based studies found decreased rates of intrapartum related neonatal deaths with resuscitation training (RR 0.70, 95%CI 0.59, 0.84). The evidence for basic resuscitation in community settings showed significant reductions in all-cause neonatal or perinatal mortality, ranging from 25-61%, and asphyxia specific mortality, ranging from 61-70% [68]. There is no existing evidence from randomized, controlled trials to support or refute the administration of epinephrine to reduce mortality and morbidity in apparently stillborn or extremely bradycardic newborn infant. Similarly, no randomized, controlled trial exists to address the issues of optimum dosage and route of administration of epinephrine. On the other hand, facility based resuscitation training have reported reductions in intrapartum related neonatal mortality and community based resuscitation training found reduction in all cause and asphyxia related neonatal mortality.

### Neonatal infection management

#### Presumptive antibiotic therapy for the newborn at risk of bacterial infection

Early onset of bacterial infection is an important cause of morbidity and mortality in newborn infants. Various factors that increase the risk of neonatal infection have been identified. It is unclear whether asymptomatic newborn infants born to mothers with one or more of these risk factors should receive antibiotics prophylactically rather than selectively if only clinical or microbiological evidence of sepsis emerges.

A review [69] identified two small trials undertaken in the 1970s. Both trials had methodological weaknesses and there was no evidence of an effect on any on these outcomes. Therefore, a large randomized controlled trial is needed in asymptomatic term infants born to mothers with risk factors for infection in their babies, which compares the effect of prophylactic versus selective antibiotics on morbidity, mortality and costs.

#### Case management of neonatal sepsis, meningitis and pneumonia

Neonatal sepsis is defined as a clinical syndrome of bacteremia with systemic signs and symptoms of infection in the first 4 weeks of life. Infection remains a major cause of illness and death in the neonatal period [70,71]. Treatment is initially with ampicillin plus either gentamicin or cefotaxime, narrowed to organism-specific drugs as soon as possible. Neonatal pneumonia is lung infection in a neonate and its signs may be limited to respiratory distress or progress to shock and death. Diagnosis is by clinical and laboratory evaluation for sepsis. Both pneumonia and neonatal sepsis can be treated in hospital or community setting.

For early onset neonatal sepsis (<48 hours), monotherapy versus combination therapy showed a decrease in mmortality in the first 28 days of life (RR 0.75; 95 % CI: 0.19, 2.90) [72]. For late onset neonatal sepsis, Beta-lactam antibiotics versus a combination of beta-lactam plus aminoglycoside decreased mortality prior to discharge (RR 0.17; 95% CI: 0.01, 3.23); and also a reduction in treatment failure (RR 0.17; 95 % CI: 0.01, 3.23) [73]. The case-management for pneumonia showed reduction in total mortality by 27% (95% CI 18,35%) and pneumonia specific mortality by 42% (22-57%) [74]. Similar findings were reported in the relatively recent review which reported 25% reduction in all-cause neonatal mortality (RR 0.75 95% CI 0.64, 0.89) and 42% reductions in pneumonia-specific mortality (RR 0.58 95% CI 0.41, 0.82) [75]. However, two studies evaluating community-based neonatal care packages including injectable antibiotics found 44% and 34% reductions in neonatal mortality (RR 0.56, 95% CI 0.41,0.77; RR 0.66, 95% CI 0.47, 0.93 respectively), but the interpretation of these results is complicated by co-interventions [75].Similar findings were reported by Bhutta et al. in a review on community-based management of neonatal sepsis; the review reported 27% reduction in all-cause neonatal mortality (95% CI: 18, 35%), and 42% reduction in pneumonia specific mortality (95% CI: 22,57%) [76]. On the basis of available evidence it can be concluded that antibiotics have a clear role in reducing neonatal mortality in LMICs and can be effectively administered at homes via trained health workers as well as for hospital-based management of neonatal sepsis.

### Interventions for small and ill babies

#### Kangaroo mother care for preterm babies

Preterm birth (before 37 completed weeks of gestation) is the most important direct cause of neonatal mortality and it accounts for an estimated 27% of the four million neonatal deaths every year [4,77]. In the early 1970s, motivated by problems arising from shortage of incubators and also the impact of mother and newborn separation, Colombian paediatrician Edgar Ray developed a technologically simple method later named Kangaroo Mother Care (KMC). Acceptance of the KMC method is increasingly widespread and it is considered equivalent to conventional neonatal care for stable preterm infants and more parent and baby friendly [78].

KMC led to a decrease in neonatal mortality (RR 0.49; 95% CI: 0.31, 0.77) [79] (RR 0.68; 95% CI: 0.48, 0.96) [80], as well as severe morbidity (RR 0.34; 95% CI: 0.18, 0.65) [79], (RR 0.57; 95% CI: 0.40, 0.80) [80]. This evidence is sufficient to recommend the routine use of KMC in facilities for babies <2000 g at birth. The potential effect of KMC is greatest in LMICs, where other options for care of preterm babies remain limited with few neonatal care facilities, mainly in distant referral hospitals and those that are often understaffed and ill-equipped.

#### Extra support for feeding the small and preterm baby

The composition of weight gained by the fetus varies with gestational age. About 80% of all weight gained between 24 and 28 weeks of gestation is water, but this proportion decreases to about 60% between 36 and 40 [81] weeks. It is usual clinical practice therefore to provide infants weighing <1500 g with about 80ml/kg for the first day of life and increase fluids by about 10-15ml/kg/day to a maximum of 160ml/kg/day by the end of the first week of life. Similarly, LBW infants >1500 g are usually given about 60ml/kg for the first day of life and the fluid intake is increased by about 15-20ml/kg/day to a maximum of 160ml/kg/day by the end of the first week of life [81-83].

A meta-analysis of studies comparing restricted with liberal fluid regimens demonstrated that restricted fluid regimens were found to be associated with a lower risk of patent ductus arteriosus (RR 0.40; 95% CI: 0.26, 0.63), necrotizing enterocolitis (RR 0.30; 95% CI: 0.13, 0.71), and death (RR 0.52; 95% CI: 0.28, 0.96) [84]. LBW babies, who are often due to preterm births, account for significant complications and poor health during infancy. According to studies included in this review, breastfeeding is the best option for LBW infants, unless unavailable, whereby donor human milk is the next best choice. Supplementation of breast milk with calcium and phosphorus is also recommended for babies with birth weight less than 1500g. Nutritional supplements, which are given separately from breast milk, available as single vitamin preparations (vitamin A, vitamin D, vitamin K) or single mineral preparations (iron, zinc, calcium and phosphorus) are also beneficial (Paper 4, vitamin A supplementation in children).

#### Prophylactic use of synthetic surfactant

Pulmonary surfactant is a substance produced by the lung alveoli and prevents the lungs from collapsing during expiration by decreasing surface tension. It is noted that babies who are born before 30 weeks, approximately 60% of them will develop respiratory distress syndrome (RDS) because lung maturity is directly proportional to the level of surfactant that is present in the newborn [85].

A review evaluated outcomes in infants given synthetic surfactant (pre-ventilatory or post-ventilatory) compared with control treatment which consisted of intratracheal administration of normal saline or air placebo. The different types of synthetic surfactant used in these trials were DPPC plus high density lipoprotein, powdered DPPC and phosphatidylglycerol and DPPC plus phosphatidylglycerol in saline. Studies also compared surfactant [86] given as either multiple or single doses to infants who were either premature (<30weeks gestation or <1250g), at risk of respiratory distress syndrome or premature infants with established respiratory distress syndrome requiring assisted ventilation. Prophylactic synthetic surfactant versus normal saline or placebo decreased rates of pneumothorax (RR 0.67; 95% CI: 0.50, 0.90); neonatal mortality (RR 0.70; 95% CI: 0.58, 0.85); pulmonary interstitial emphysema (RR 0.68; 95% CI: 0.50, 0.93); and mortality at 1 year (RR 0.83; 95% CI: 0.70, 0.98) [86]. Multiple versus single dose of surfactant decreased rates of pneumothorax (RR 0.70; 95%CI: 0.52, 0.94); pulmonary interstitial emphysema (RR 0.62; 95% CI: 0.54, 0.71), mortality (RR 0.59; 95% CI: 0.44, 0.78), and necrotizing enterocolitis (RR 0.18; 95% CI: 0.07, 0.44). No significant changes were seen in rates of patent ductus arteriosus or intraventricular haemorrhage [86]. Infants who underwent prophylactic administration of synthetic surfactant had a decreased risk of pneumothorax, pulmonary interstitial emphysema and neonatal mortality. Also infants who were given synthetic surfactant showed an increased risk of developing patent ductus arteriosus and pulmonary haemorrhage. Rresults also show that multiple doses of surfactant as prevention or treatment resulted in a statistical significance reduction in the incidence of pneumothorax, mortality and necrotizing enterocolitis.

#### Therapeutic surfactant use for respiratory distress syndrome

Even though surfactant therapy has shown to improve outcomes for premature infants with RDS it is important to note that this therapy should not be used as an alternative to prolonging premature deliveries in order to increase lung maturity, or giving mothers antenatal corticosteroids with the aim to prevent RDS from developing [87-91].

A meta-analysis supports decrease in the risk of pneumothorax (RR 0.64, 95% CI 0.55,0.76), pulmonary interstitial emphysema (RR 0.62, 95% CI 0.54, 0.71), patent ductus arteriosus (RR 0.90, 95% CI 0.84, 0.97), intraventricular haemorrhage (RR risk 0.88, 95% CI 0.77, 0.99), broncho-pulmonary dysplasia (RR 0.75, 95% CI 0.61, 0.92), neonatal mortality (RR 0.73, 95% CI 0.61, 0.88), broncho-pulmonary dysplasia or death at 28 days (RR 0.73, 95% CI 0.65, 0.83), mortality prior to hospital discharge (RR 0.79,95% CI 0.68, 0.92) and mortality during the first year of life (RR 0.80, 95% CI 0.69, 0.94). Treatment with synthetic surfactant increases the risk of apnea of prematurity (RR 1.20, 95% CI 1.09, 1.31) [92]. Early versus delayed selective surfactant treatment decreased chronic lung disease rates (typical RR 0.69; 95% CI: 0.55, 0.86; typical Risk Difference (RD) -0.04; 95% CI: -0.06 , -0.01); acute lung injury including a decreased risk of pneumothorax (typical RR 0.69; 95% CI: 0.59, 0.82); pulmonary interstitial emphysema (typical RR 0.60; 95% CI: 0.41, 0.89) and broncho-pulmonary dysplasia (BPD) or death at 28 days was also evident (typical RR 0.94; 95% CI: 0.88, 1.00) [93]. Existing evidence shows that giving synthetic surfactant to infants with established RDS improves clinical outcome. Infants who are treated with synthetic surfactant were found to have a decreased risk of pneumothorax, pulmonary interstitial emphysema, intraventricular haemorrhage, and Broncho pulmonary dysplasia. There was also a reduction in the incidence of neonatal mortality, mortality prior to hospital discharge and at 1 year of age. Infants who received synthetic surfactant treatment for established RDS also had an increased risk of apnea of prematurity. All results that are mentioned in this section reached statistical significance upon analysis. The results in the review of Soll 2009 et al. were for both prevention and treatment of respiratory distress syndrome. They showed that multiple doses of surfactant resulted in a decreased risk pneumothorax, mortality and necrotizing enterocolitis.

#### Continuous positive airway pressure (CPAP) to manage pre-term babies with respiratory distress syndrome

Apnea of prematurity, which is commonly seen in infants who are born before 34 weeks of gestation, is defined as a pause in breathing for greater than twenty seconds or an apneic event that is less than twenty seconds but is associated with bradycardia and/or cyanosis [94,95]. Continuous positive airway pressure (CPAP), has shown to be effective without long term effects; however, this has not been fully observed. Another treatment option is with ventilation (NIPPV, nasal intermittent positive pressure ventilation) which is delivered through the nasal route, and this also has seen to be effective in preterm infants whose apnea is frequent and severe [96]. As with the other treatment options there are some drawbacks with the use of NIPPV also, it has been noted that providing ventilation via nasal prongs is associated with an increased risk of gastrointestinal perforation [97].

A meta-analysis demonstrates that high frequency positive pressure ventilation (HFPPV) compared to conventional ventilation (CMV) was associated with a reduction in the risk of air leak (RR 0.69, 95% CI 0.51, 0.93). Assist control ventilation (ACV) or synchronized intermittent mandatory ventilation (SIMV) compared to CMV was associated with a shorter duration of ventilation (WMD -34.8 hours, 95% CI -62.1, -7.4). ACV compared to SIMV was associated with a trend to a shorter duration of weaning (WMD -42.4 hours, 95% CI -94.4, 9.6). Neither HFPPV nor triggered ventilation was associated with a significant reduction in the incidence of BPD [98]. NIPPV vs. NCPAP decreased rates of failure of therapy (intubation) (RR 0.30; 95%CI: 0.01, 6.84), rate of apnea (events/hr) (MD -0.10 events/hr; 95%CI: -0.53, 0.33) and change in rate of apnea (events/hr) (MD -1.19 events/hr; 95%CI: -2.31, -0.07). NIPPV when compared to NCPAP may be a useful method in preterm infants with apnea [99]. Continuous distending pressure (CDP), on the other hand, is found to be associated with a lower rate of failed treatment (death or use of assisted ventilation) (RR 0.65 95% CI 0.52, 0.81), overall mortality (RR 0.52 95% CI 0.32, 0.87), and mortality in infants with birth weights above 1500 g (RR 0.24 95% CI 0.07, 0.84). The use of CDP is associated with an increased rate of pneumothorax (RR 2.64 95% CI 1.39, 5.04) [100]. A review by Greenough et al. [98] shows that there are more benefits in treating neonates with HFPPV or triggered ventilation compared to conventional ventilation. There was a reduction in both air leaks and duration of ventilation. Even though benefits were documented in this review, we encourage that more trials are done in order to determine the effectiveness of ventilation, along with other benefits or long term risk associated with these methods.

#### Management of newborns with jaundice

Extremely high levels of bilirubin (severe jaundice) can lead to brain damage. Severe jaundice in newborns can occur as a result of a variety of causes including rhesus hemolytic disease, ABO incompatibility, atypical antibodies etc. Removal of blood from the affected infant and replacing with fresh blood from the blood bank (exchange transfusion) is used as a treatment for severe jaundice in newborn infants.

Phototherapy is used to treat newborn infants with hyperbilirubinaemia. Fibreoptic phototherapy is a new mode of phototherapy which is reported to lower serum bilirubin (SBR) while minimizing disruption of normal infant care. Fibreoptic phototherapy was found to be more effective at lowering SBR (serum bilirubin) than no treatment but less effective than conventional phototherapy. Fibreoptic phototherapy was equally as effective as conventional phototherapy in preterm infants and when two fibreoptic devices were used simultaneously (change in SBR after 24 hours of treatment: (WMD 1.7%; 95%CI: -2.65, 6.05) and change in SBR per day over whole treatment period: (WMD 2.82%; 95%CI: -1.84, 7.48 respectively). A combination of fibreoptic and conventional phototherapy was more effective than conventional phototherapy alone (duration of phototherapy): (WMD -12.51 hr; 95%CI: -16.00, -9.02, meta-analysis affected by heterogeneity) [101].

Fibreoptic phototherapy has a place in the management of neonatal hyperbilirubinaemia. It is probably a safe alternative to conventional phototherapy in term infants with physiological jaundice.

Double volume exchange transfusion is commonly used in newborns with severe jaundice in order to prevent kernicterus and other toxicity related to hyperbilirubinemia. A single trial fulfilling the criteria for inclusion identified 20 full term babies requiring exchange transfusion for hemolytic jaundice due to ABO incompatibility. These infants were randomly allocated to receive single or double volume exchange transfusion. Total bilirubin levels immediately after exchange transfusion were not significantly different in either group. There was insufficient evidence to support or refute the use of single volume exchange transfusion as opposed to double volume exchange transfusion in jaundiced newborns [102].

## Discussion

This paper summarized all the essential childbirth and postnatal interventions for improved maternal and newborn health. The interventions were preventive and therapeutic aimed at preventing illnesses and improve survival of mothers and babies. The importance of these interventions has been addressed in previous publications [103-106]. However this review further underscored their standing by collating the existing evidence to assist health professionals and policy makers to reduce the existing burden of maternal and neonatal morbidity and mortality.

Among the childbirth interventions, the review highlighted the role of injecting oxytocin immediately after childbirth for the prevention and management of postpartum hemorrhage, which is lethal and is responsible for large number of maternal deaths if unattended. Similarly, practicing good hygiene and recognition of early signs of infection after childbirth can be instrumental in saving lives. Detection and management of pre-eclampsia and use of magnesium sulphate can lower a woman’s risk of developing eclampsia and other life-threatening complications. For postnatal interventions, care of the neonate and mother in the hours following the delivery is vital and must be adequate to prevent the high risk of mortality and morbidity associated with it. The benefits of breastfeeding have been well documented; however the timing of start of breastfeeding is very important, with studies suggesting much greater benefits of early vs. late feeding [56]. Hygienic care of cord and skin of the baby after delivery for lowering the risk of infections have the potential to reduce neonatal deaths. Neonatal resuscitation is vital for preventing neonatal deaths due to birth asphyxia. Babies with respiratory problems should be assessed and administered surfactant or other ventilatory assistance such as CPAP immediately. Babies with LBW are advised to receive thermal care, as well as KMC along with appropriate extra support for feeding. Childbirth and postnatal care should not be limited to a definite period whereby only the current health status of both mother and child are addressed but should also link the mother to family-planning services and the baby to child health care which will cater to the future needs of both child and mother [107]. The focus of delivering effective postnatal care must be on the timely delivery of intervention packages, i.e. on the first day after birth.

Childbirth and postnatal care faces many challenges in LMICs whereby establishing contact between the pregnant mother, postpartum mother, or neonate is difficult since home births and seclusion may be a community practice or tradition. However, the importance of community based delivery strategies to increase access to needed care is paramount to bringing about a positive change in the developing countries. Although not all the interventions discussed in this paper can be delivered in community, training community midwives and detailing proper linkages with local health facilities is very vital. Referrals from community to health facilities for interventions such as, management of postpartum hemorrhage and cesarean sections is very critical. Therefore, training midwives and follow-ups through home visits may assist in increasing the coverage and improving the quality of home-based postnatal care services for mothers and newborns. This goal can be achieved through increased utilization of basic postnatal services by mothers and newborns, increased identification and referral of post-partum women and newborns with health problems to health care facilities, and provision of quality home based postnatal care for mothers and newborns [108].

This paper is a comprehensive and through summary of essential childbirth and postnatal interventions for health care practitioners and decision makers who can integrate these interventions into health system for improved maternal and child health. If implemented properly to target a wider audience, keeping in mind the barriers to implementation, then major progress may be made in meeting the MDG’s and higher maternal and neonatal maternal mortality would be ceased. The strength of this paper is that it included and summarized evidence from all the recent Cochrane and non-Cochrane reviews on childbirth and postnatal interventions for mothers and newborns. However, at times the quality of all included studies within the review could not be ensured which limited the quality of the data obtained.

## Conclusion

Maternal and newborn health exists in a synergistic relation. Most maternal deaths are avoidable provided timely and adequate delivery of health-care solutions to prevent or manage complications. An access to antenatal care in pregnancy, skilled care during childbirth, and care and support in the weeks after childbirth should be provided to all women. The review discussed all childbirth and postnatal interventions which have an impact on reducing maternal, neonatal mortality and which are suitable for delivery in LMICs. The implementations of discussed interventions promise a much needed improvement is maternal and neonatal outcomes around the world. However, some of these interventions must be prioritized over others depending on the clinical indications and keeping in view the limited resources in developing countries. Timely provision of these interventions holds unparalleled significance, particularly those that are delivered during and immediately after childbirth, in places where majority of the births occur, at home.

## Competing interests

We do not have any financial or non-financial competing interests for this review.

## Peer review

The reviewer reports for this article can be found in Additional File 1.

## Supplementary Material

Additional file 1 Peer review reports. Click here for file

## Declarations

This article has been published as part of *Reproductive Health* Volume 11 Supplement 1, 2014: Essential intervention for maternal, newborn and child health. The full contents of the supplement are available online at http://www.reproductive-health-journal.com/supplements/11/S1. Publication charges for this collection were funded by the Partnership for Maternal, Newborn & Child Health (PMNCH).
