# Supplementary material for: Essential childbirth and postnatal interventions for improved maternal and neonatal health
Source: Reprod Health. 2014 Aug 21;11(Suppl 1):S3. doi: 10.1186/1742-4755-11-S1-S3 (PMC4145857; doi:10.1186/1742-4755-11-S1-S3)
Supplement: Additional file 1 — Peer review reports. [file 1742-4755-11-S1-S3-S1.pdf]

Referee's comments to the authors– this sheet WILL be seen by the author(s) and published with the article

Title	**Essential childbirth and postnatal interventions for improved maternal and neonatal health**
Author(s)	Rehana A Salam,TarabMansoor, Dania Mallick, Zohra S Lassi, Jai K Das, Zulfiqar A Bhutta
Referee's name	Jennifer Requejo

When assessing the work, please consider the following points, where applicable:

1. Is the question posed by the authors new and well defined?
2. Are the methods appropriate and well described, and are sufficient details provided to replicate the work?
3. Are the data sound and well controlled?
4. Does the manuscript adhere to the relevant standards for reporting and data deposition?
5. Are the discussion and conclusions well balanced and adequately supported by the data?
6. Do the title and abstract accurately convey what has been found?
7. Is the writing acceptable?

Please make your report as constructive and detailed as possible in your comments so that authors have the opportunity to overcome any serious deficiencies that you find and please also divide your comments into the following categories:

- Major Compulsory Revisions (which the author must respond to before a decision on publication can be reached)
- Minor Essential Revisions (such as missing labels on figures, or the wrong use of a term, which the author can be trusted to correct)
- Discretionary Revisions (which are recommendations for improvement but which the author can choose to ignore)

Where possible please supply references to substantiate your comments.

When referring to the manuscript please provide specific page and paragraph citations where appropriate.

General comments:

This paper presents a review of some essential interventions delivered around the time of birth and during the postnatal period for both mother and baby. It is a timely paper given the short time frame to the MDG deadline, and the need to scale-up proven maternal and newborn interventions. Although the paper provides a lot of excellent detail on available evidence concerning specific life-saving interventions, it is unclear what the significance is of the review for guiding clinical practice, planning/prioritization purposes, or for future research.

The introduction does not clearly spell out the structure of the paper (i.e., the flow of the paper and how each section fits together) and there is no readily identifiable methods section or clarity on what 'essential' interventions are being reviewed, the sources used for the review, and why these particular interventions only are being reviewed. Also lacking is some sense of how these interventions can be delivered – for example, how can this review help inform efforts to clarify the timing and content of postnatal care services for both women and their babies? Another option could be to structure the paper around interventions related to major causes of maternal and child deaths, with some ideas on how these interventions should ideally be delivered through the major platforms (ANC, SBA, and PNC).

The authors emphasize in the introduction that the continued high levels of maternal and newborn deaths mainly in low-resource countries is a reflection of a lack of facilities. This seems to overlook the importance of

community based delivery strategies to increase access to needed care. Can the authors address this? And, there is a nod to the interlinkages of maternal and newborn health in the conclusion, but this is not made evident in the scope of the review. Similarly, there are references to maternal health as one of the 8 MDGs (would be good to note that it is MDG5), and might make sense to reference MDG4 as well given that MDG4 does pertain to the newborn. The conclusion talks about newborn mortality but skims over maternal health. I would suggest that the intro and conclusion be a bit more consistent in the presentation of the data on maternal and newborn health and their interconnection.

There is also a need for an editorial review of the article to simplify many of the sentences and to reduce the redundancies in the article.

Major Compulsory Revisions

1. Both the introduction and the abstract should explain why the review was undertaken, or why this compilation of evidence on a few select interventions makes a contribution to the literature.
2. The authors need to add a clear paragraph on the methods, including why this particular list of interventions is being reviewed in this paper, what the sources are for the review, and the approach used to review or compile the information.
3. The authors should add in something to the introduction that describes the organizational structure of the paper.
4. The conclusion and discussion at the end of each of the sections should contain some sort of summary or analytical synthesis about the information presented (e.g., programmatic, policy relevance, or where there are research gaps that need to be filled, how the interventions described can be combined or provided through existing delivery strategies, etc.). The conclusion notes that if these interventions were introduced, then there would be major reductions in mortality. However, there is little in the paper on how this might be accomplished and if some interventions should be prioritized over others if there are resource constraints, etc.

Referee's comments to the authors– this sheet WILL be seen by the author(s) and published with the article

Title	**Essential childbirth and postnatal interventions for improved maternal and neonatal health**
Author(s)	Rehana A Salam,TarabMansoor, Dania Mallick, Zohra S Lassi, Jai K Das, Zulfiqar A Bhutta
Referee's name	Nadeem Zuberi

When assessing the work, please consider the following points, where applicable:

1. Is the question posed by the authors new and well defined? Yes
2. Are the methods appropriate and well described, and are sufficient details provided to replicate the work? Yes
3. Are the data sound and well controlled? Yes
4. Does the manuscript adhere to the relevant standards for reporting and data deposition? Yes
5. Are the discussion and conclusions well balanced and adequately supported by the data? Yes
6. Do the title and abstract accurately convey what has been found? Yes
7. Is the writing acceptable? Yes

Please make your report as constructive and detailed as possible in your comments so that authors have the opportunity to overcome any serious deficiencies that you find and please also divide your comments into the following categories:

- Major Compulsory Revisions (which the author must respond to before a decision on publication can be reached)
- Minor Essential Revisions (such as missing labels on figures, or the wrong use of a term, which the author can be trusted to correct)
- Discretionary Revisions (which are recommendations for improvement but which the author can choose to ignore)

Where possible please supply references to substantiate your comments.

When referring to the manuscript please provide specific page and paragraph citations where appropriate.

General comments: Authors have systematically compiled the current best evidence on the subject. A well written comprehensive review.

Major compulsory revisions: None

Minor essential revisions: None

Discretionary revisions: None

Supplement Editor's comments

Please give special attention to one reviewer comment about the need of a clear statement of the main aims of the paper.
Your paper is lacking a clear organizational structure, the methods are not explained, and there are no research recommendations or clear guidelines for clinical practice presented. Please focus in these concerns in your revised manuscript.
Your paper needs an editorial overhaul to remove redundancies and to tighten the English.
The article must read independently of each other: although the articles will be published together with a supplement, it's important that each article can be read and understood on its own.

Authors' response to reviewers

General comments:

This paper presents a review of some essential interventions delivered around the time of birth and during the postnatal period for both mother and baby. It is a timely paper given the short time frame to the MDG deadline, and the need to scale-up proven maternal and newborn interventions. Although the paper provides a lot of excellent detail on available evidence concerning specific life-saving interventions, it is unclear what the significance is of the review for guiding clinical practice, planning/prioritization purposes, or for future research.Added this in Intro and discussion now.

The introduction does not clearly spell out the structure of the paper (i.e., the flow of the paper and how each section fits together) and there is no readily identifiable methods section or clarity on what 'essential' interventions are being reviewed, the sources used for the review, and why these particular interventions only are being reviewed. Also lacking is some sense of how these interventions can be delivered – for example, how can this review help inform efforts to clarify the timing and content of postnatal care services for both women and their babies? Another option could be to structure the paper around interventions related to major causes of maternal and child deaths, with some ideas on how these interventions should ideally be delivered through the major platforms (ANC, SBA, and PNC). Methods section added. We have used the stabndard structure of essential intervention report. However, the deliverability is now discussed in the discussion section.

The authors emphasize in the introduction that the continued high levels of maternal and newborn deaths mainly in low-resource countries is a reflection of a lack of facilities. This seems to overlook the importance of community based delivery strategies to increase access to needed care. Can the authors address this? And, there is a nod to the inter linkages of maternal and newborn health in the conclusion, but this is not made evident in the scope of the review. Similarly, there are references to maternal health as one of the 8 MDGs (would be good to note that it is MDG5), and might make sense to reference MDG4 as well given that MDG4 does pertain to the newborn. The conclusion talks about newborn mortality but skims over maternal health. I would suggest that the intro and conclusion be a bit more consistent in the presentation of the data on maternal and newborn health and their interconnection. The community based startegies for maternal and newborn health as an intervention has been discussed in detail in Paper 2. We have also added the respective MDGs with maternal and newborn health points. We have revisited conclusion as well.

There is also a need for an editorial review of the article to simplify many of the sentences and to reduce the redundancies in the article. Shortened the introduction and added discussion section. Discussion was added because it was suggested for paper 2.

Major Compulsory Revisions

1. Both the introduction and the abstract should explain why the review was undertaken, or why this compilation of evidence on a few select interventions makes a contribution to the literature. Added
2. The authors need to add a clear paragraph on the methods, including why this particular list of interventions is being reviewed in this paper, what the sources are for the review, and the approach used to review or compile the information. Added
3. The authors should add in something to the introduction that describes the organizational structure of the paper.added in last paragraph of introduction.
4. The conclusion and discussion at the end of each of the sections should contain some sort of summary or analytical synthesis about the information presented (e.g., programmatic, policy relevance, or where there are research gaps that need to be filled, how the interventions described can be combined or provided through existing delivery strategies, etc.). The conclusion notes that if these interventions were introduced, then there would be major reductions in mortality. However, there is little in the paper on how this might be accomplished and if some interventions should be prioritized over others if there are resource constraints, etc. added
